# Developing digital mental health tools for youth with diabetes: an agenda for future research

**DOI:** 10.3389/fcdhc.2023.1227332

**Published:** 2023-07-11

**Authors:** Katie M. Babbott, Anna Serlachius

**Affiliations:** Department of Psychological Medicine, University of Auckland, Auckland, New Zealand

**Keywords:** diabetes, digital mental health, eHealth, psychological support, youth

## Abstract

Youth living with diabetes face a concurrent challenge: managing a chronic health condition and managing the psychosocial and developmental changes that are characteristic of adolescence and young adulthood. Despite these unique challenges, psychological support is often difficult for youth with diabetes to access due to a lack of trained mental health professionals and other resource constraints. Digital wellbeing tools offer the potential to improve access to psychological support for this population. However, very few digital wellbeing tools exist for youth with diabetes. Of those that do exist, very few are evidence-based therapies, undermining their contribution to the field. Given the increasing global prevalence of diabetes in young people, the support necessitated by the challenges experienced by this population is not always accessible in a face-to-face setting and cannot be effectively scaled to meet demand. To support the health and wellbeing of youth with diabetes, there is a clear need to develop digital interventions that are widely accessible to users, but, more saliently, grounded in empirical evidence that supports their efficacy. Thus, the purpose of this paper is to offer an agenda for future research, including insights into which psychological techniques and behavioral change theories may be a good conceptual fit for digital mental health interventions, and how these tools may be best developed and utilized by the individuals that need them. Scalable, evidence-based wellbeing tools for this population are urgently required to improve psychological outcomes, and potentially, improve the equity of service access.

## 1 Introduction

The self-management requirements related to type 1 diabetes (T1D) and type 2 diabetes (T2D) are well-documented challenges for individuals worldwide (1–3). In particular, youth living with diabetes face the challenge of simultaneously managing the psychosocial and developmental changes that are characteristic of adolescence and young adulthood, as well as the stringent medical and self-care requirements of their condition (4). Among youth, both T1D and T2D are often comorbid with significant psychological concerns (5, 6). Research suggests that as many as one in three youth with T1D and T2D experience substantial distress specifically related to their diagnosis (7, 8), but that routine monitoring of psychosocial outcomes and collaborative, psychosocial support can improve psychological well-being and diabetes outcomes (9–11). However, given the increasing global prevalence of both T1D and T2D in young people (12–15), the support required by this population is not always accessible in a face-to-face format and cannot be adequately scaled to meet rising demand. Unfortunately, very few digital well-being tools currently exist to equitably support the unique well-being needs of this population (16). Of those that do exist, even fewer are developed for youth with T2D (10, 16). Thus, in advancing and promoting the mental health and well-being of youth living with diabetes, continued research is needed to establish how theoretically founded, evidence-based interventions can be designed to ensure that youth with diabetes requiring psychological support are able to access it equitably, and in a timely and affordable manner.

## 2 Digital health interventions: can they offer a scalable solution?

A rapidly growing body of work suggests that digital interventions may offer an efficacious solution to the unmet needs of youth living with diabetes. Broadly, digital well-being interventions are defined as any intervention which is offered within a digital ecosystem, including online or web-based platforms, smartphone applications (‘apps’), serious games, or augmented or virtual reality programs (17, 18). To date, digital well-being interventions have effectively reduced existing barriers to psychological support in both non-clinical and clinical populations, such as cost, stigma, and accessibility issues (19–21), and they are growing in popularity among youth, for whom low rates of healthcare access exist (22, 23).

Device ownership and access to digital technologies is almost universal among youth, with 99% of adolescent and young adult participants in a 2022 study in Australia reporting they own a device with internet connectivity, and 63% of the sample reporting that they use this device more than once an hour (24). Further, consumers (both clinicians and youth end users) are consistent in reporting that digital interventions are sought after (23). They are easily scalable and widely accessible and serve as a useful adjunct to specialist and primary care and can also be efficacious as standalone tools (17). Such scalability and accessibility suggests potential for global use; that is, there is the potential for effective digital interventions be translated and made available globally, also in low- and middle-income countries (25).

Digital interventions may be especially useful for youth diagnosed with T2D, who often have equivalent or even greater needs for psychosocial support, but are not as closely monitored by treatment teams and clinicians in comparison to youth with T1D (26, 27).

To date, many digital interventions have largely been developed for adult populations, and not specifically to meet the unique needs of individuals living with chronic health conditions such as diabetes. Of those that do exist, very few are evidence-based therapies or based on behavior change theories, undermining their contribution to the field (16). Thus, in refining ongoing research pursuits there is a clear need to develop digital interventions that are widely accessible to users, but, more saliently, grounded in empirical evidence that supports their efficacy. An agenda for future research is offered below, including insights into which psychological techniques and behavioral change theories may be a good conceptual fit for youth with diabetes when delivered as digital mental health tools, and how these tools may be best utilized by the individuals that need them.

## 3 Research agenda: where to from here?

### 3.1 Which theoretical framework?

Given that youth are digital natives and are typically very comfortable using technology, digital interventions offer a promising equitable alternative to existing face-to-face interventional strategies. However, systematically reviewed research which was specific to youth with T1D highlighted mixed findings in terms of the efficacy of existing digital interventions designed to improve mental health and wellbeing (16). Of note, a key concern noted by this review was the lack of a robust theoretical framework underpinning interventional design, and an absence of evidence-based techniques (such as those grounded in a theoretical approach such as CBT). This methodological variety makes it challenging to identify the most effective frameworks and formats for digital interventions, and for whom they may work best.

When considering which theoretical approaches are best suited for digital mental health tools for youth with diabetes, an important starting point is considering the evidence of face-to-face psychological interventions for this population. In face-to-face interventions for youth with diabetes, coping skills training, CBT-based interventions, and family-based interventions such as Behavioral Family Systems Therapy have all demonstrated efficacy for improving psychological outcomes (28, 29). Preliminary evidence also exists for some third-wave cognitive therapies (such as Acceptance and Commitment Therapy and Mindfulness-based interventions) in terms of being acceptable (30) and reducing diabetes distress and depressive symptoms (31) in youth with diabetes, and improving both glycaemic outcomes and psychological wellbeing in adults with diabetes (32). A key limitation of the existing evidence-based psychosocial interventions (whether delivered face-to-face or online) is the lack of interventions conducted in youth with T2D (16, 29).

### 3.2 How can we optimize digital delivery?

The extensive face-to-face interventions highlights the ways in which psychoeducational programs can effectively foster self-efficacy, coping skills, family functioning, and goal setting among youth with diabetes, leading to improvements in psychological outcomes (33–35). How such interventions (and the frameworks that underlie them) translate to the digital world is not yet well-understood; existing digital health interventions for youth with diabetes face challenges with participant drop-out, largely related to such interventions not meeting the needs of users (36). In advancing future research, translating findings from established evidence-based face-to-face interventional strategies to the digital ecosystem will be reliant on understanding what works best for the target demographic, and what features youth living with diabetes are looking for in the digital technologies they consume. Though it appears that digital interventions with in-person elements (such as peer support or clinician oversight) are linked to lower participant drop-out, there is limited data on the factors which can reduce attrition and support uptake among youth living with diabetes (37).

Further, filling the notable gap in the literature for youth with T2D in particular is an important area of focus for ongoing study. Going forward, utilizing a co-design or a user-centered design strategy is likely an effective methodological decision to ensure digital interventions are developed to be feasible and acceptable for all youth (38) including youth with both T1D and T2D. Though co-design can enhance engagement and maintain participation (39), it is somewhat limited in research among youth (40). Systematically reviewed work offers evidence of an increased use of co-design for youth digital health apps; however, in the interest of best practice, the field needs to move from a place of consultation (with youth and end-users) to a place of collaboration (41).

To date, mixed methods co-design research is limited, but qualitative data conducted by our group has indicated that key features that youth with diabetes want in digital mental health interventions is the ability to connect with their peers, diabetes-specific content, and a focus on both emotional and physical wellbeing (42, 43). Though these qualitative studies were limited to youth with T1D, it highlights that in both face-to-face (44) and digital interventions, the element of social connection, and, more specifically, peer-to-peer support, is important. This prompts further questions about how existing group-based interventions can be adapted for the digital world without the loss of the interventional element of connection. Additionally, questions remain regarding how such connection can be facilitated in a way that is ethical and safe, and without placing the onus of facilitating peer-to-peer contact onto already stretched clinicians. Though peer-to-peer support is widely considered a beneficial element of interventions, a number of barriers exist to embedding this support, including the informal nature of such interactions creating an environment within which misinformation can spread and interpersonal challenges can emerge (45). Establishing ways in which risk and potential benefit can be balanced is a key area of focus for ongoing work.

Another important consideration when developing digital mental health tools for youth with diabetes is user engagement. Prior research exploring user engagement of digital mental health interventions in the general population suggests that uptake and sustained use of digital mental health tools remains low and reflects the biggest barrier in regards to implementation and dissemination of effective tools (46). A similar concern has been found for eHealth interventions for diabetes, where lower engagement is a frequent problem among youth, despite the fact that higher participation is associated with better outcomes (47).

Other promising approaches for tackling low engagement of digital tools include brief digital mental health interventions, which have demonstrated improved retention in adolescents and have also demonstrated improvements in mental health outcomes in non-clinical samples of adolescents and young adults (48) as well as adults living with chronic health conditions (49). What remains to be well-understood is which platforms are preferred by users for these adaptations and how these adaptations can be integrated within regular care.

### 3.3 The use of digital interventions in routine care

Guided digital mental health interventions (which offer some human guidance) have been shown to be more effective than self-guided tools for improving depression in the wider digital health literature (50). However, unlike self-guided digital tools developed for the general population, digital mental health tools for youth with diabetes would ideally be incorporated into routine care, in order to augment existing psychosocial support, representing an ideal interventional strategy (51, 52). It is therefore important to emphasize that digital mental health tools for youth with diabetes should support and augment existing psychological services, not replace them.

A final point worth noting is that of the role of digital tools in equity enhancing care. Digital tools are uniquely positioned to offer access to healthcare that may otherwise have been difficult for people from marginalized communities to access (53). However, such tools must be developed with digital inclusion in mind. That is, ways in which digital interventions can be developed to be accessible, affordable, relevant and usable for the target population must be at the forefront of development priorities, with considerations also made for health and digital literacy (39). For diabetes in particular, digital tools could be an effective way of routine monitoring, screening, and communicating data with healthcare providers. However, if such tools are not designed and developed with careful thought given to the factors that may prevent users from inclusion, access and engagement, these tools have the potential to expand inequity rather than reduce it (54). A useful starting point is to consider the Framework for Digital Health Equity (54), which outlines differing domains of influence including the digital environment and considerations such as technology access, community infrastructure and interdependence (e.g. shared devices). Therefore, attention must be paid to barriers at the individual, community, service, and policy levels (55). For example, ensuring that co-design practices are inclusive and representative of individuals from a wide range of cultural, ethnic, and socioeconomic backgrounds will ensure that gaps in information regarding health data, deliverables, and interventional design are able to be filled (56) and interventions are able to meet the needs of users, and uptake is easy and equitable.

Finally, in accurately evaluating digital mental health tools for youth with diabetes and offering data to healthcare professionals involved in routine diabetes management and care, thought must be given to primary outcomes of interest. To date, evaluating the efficacy of psychosocial interventions in diabetes has been unduly focused on glycaemic metrics, largely HbA1c (33). Although physiological outcomes are not unimportant, measuring clinical psychological outcomes (such as depression or anxiety), diabetes-specific psychological outcomes (such as diabetes-related distress), and lastly strength-based outcomes (such as emotional wellbeing, resilience, and empowerment) are arguably more important when evaluating interventions developed to improve psychosocial outcomes. Lastly, all of these measures help to facilitate the discussion of psychological health and wellbeing within the clinical team as an integral part of routine care, thereby helping to normalize and de-stigmatize the discussion of mental health in diabetes.

## 4 Conclusions and clinical implications

To support the psychological wellbeing of youth with diabetes, scalable, measurable, and evidence-based digital wellbeing tools for this population are urgently required to improve psychological outcomes, and potentially, improve the equity of service access. By developing digital wellbeing interventions with input from youth with diabetes, their caregivers, and clinicians, combined with integrated care alongside face-to-face psychosocial services, broader access to psychological services will be available to youth living with diabetes.

## Data availability statement

The original contributions presented in the study are included in the article/supplementary material. Further inquiries can be directed to the corresponding author.

## Author contributions

AS came up with the concept for the article. AS and KB both drafted and revised the article. All authors contributed to the article and approved the submitted version.
